# The dampening effect of employees’ future orientation on cyberloafing behaviors: the mediating role of self-control

**DOI:** 10.3389/fpsyg.2015.01482

**Published:** 2015-09-29

**Authors:** Heyun Zhang, Huanhuan Zhao, Jingxuan Liu, Yan Xu, Hui Lu

**Affiliations:** ^1^School of Psychology, Beijing Normal UniversityBeijing, China; ^2^Department of Educational Psychology, Purdue University, West LafayetteIN, USA

**Keywords:** future orientation, self-control, cyberloafing, mediation effect, employees

## Abstract

Previous studies on reducing employees’ cyberloafing behaviors have primarily examined the external control factors but seldomly taken individual internal subjective factors into consideration. Future orientation, an important individual factor, is defined as the extent to which one plans for future time and considers future consequences of one’s current behavior. To explore further whether and how employees’ future orientation can dampen their cyberloafing behaviors, two studies were conducted to examine the relationship between employees’ future orientation and cyberloafing behaviors. The mediation effect of employees’ objective and subjective self-control between them was also examined. In Study 1, a set of questionnaires was completed, and the results revealed that the relationship between employees’ future orientation and cyberloafing behaviors was negative, and objective self-control mediated the relationship. Next, we conducted a priming experiment (Study 2) to examine the causal relationship and psychological mechanism between employees’ future orientation and cyberloafing behaviors. The results demonstrated that employees’ future-orientation dampened their attitudes and intentions to engage in cyberloafing, and subjective self-control mediated this dampening effect. Theoretical and practical implications of these findings are also discussed.

## Introduction

The Internet is a double-edged sword, providing not only great convenience for employees and companies but also introduces many unexpected problems. Employees’ cyberloafing is an outstanding problem, which is a new form of counterproductive work behaviors (CWB). Cyberloafing means that employees use their companies’ Internet access for non-work-related purposes during working hours (Lim, 2002; Lim and Chen, 2012), which has the following characteristics: first, it is not as visible as other loafing behaviors (Wagner et al., 2012). Many forms of CWB, including taking long lunches, chatting with coworkers, coming late, and leaving early are easily identified as loafing behaviors. However, cyberloafing does not require a person to be physically absent from the office for a long time, and employees can be engaged in cyberloafing behaviors even without leaving their desks. So it is difficult to discern employees’ cyberloafing behaviors by observation. Second, it causes much more serious harm on employees’ productivity (Lim and Chen, 2012), and decreases the benefits of enterprises (Nair, 2005; Jia et al., 2013). Some studies have revealed that cyberloafing behaviors extensively existed in workplace (Lim and Teo, 2005; Blanchard and Henle, 2008). It does not only decrease employees’ productivity by 30–40% (Conlin, 2000; Lim, 2005; Lim and Teo, 2005), but also cause a large economic loss of enterprises annually (Malachowski, 2005). Sometimes, employees’ cyberloafing behaviors (e.g., downloading music, accessing pornographic sites, viewing or sending offensive material) can even put the enterprises at risk (Lichtash, 2004; Blanchard and Henle, 2008). Given these reasons, the development of strategies and methods to reduce employees’ cyberloafing behaviors has been an important and worthwhile research theme.

In addition, previous studies have investigated the external environment factors that influence employees’ cyberloafing behaviors, such as work stressors (Henle and Blanchard, 2008; RuningSawitri, 2012), organizational characteristics (Liberman et al., 2011), organization sanctions and policies (Ugrin and Pearson, 2013), organizational justice (Lim, 2002, 2005; De Lara, 2007; Henle et al., 2009), and social norms (Blanchard and Henle, 2008; Askew, 2012). Researchers suggest that employers can reduce employees’ cyberloafing behaviors by optimizing these external environment factors. In other words, employees allow external forces outside their personal control to regulate their behaviors. Nevertheless, the self-determination theory (SDT) suggests that individuals need a sense of autonomy and self-control that are conducive to enhancing their job involvement and performance (Kasser et al., 1992; Baard et al., 2004). Employees can proactively dampen their own cyberloafing behaviors. Hence, we should explore individual internal subjective factors that can influence employees’ cyberloafing behaviors. In the present research, we want to construct a mediation model to examine how to reduce employees’ cyberloafing behaviors from the perspective of individual factors.

Accordingly, the present research may have two major contributions. First, it can extend the prior work on cyberloafing behaviors by highlighting the influence of individual factors. Second, it also can provide some possible strategies and methods to reduce employees’ cyberloafing behaviors.

### Future Orientation and Cyberloafing

Individual future orientation is an important variable influencing human behaviors, even though it has been ignored within the framework of cyberloafing research. The concept of future orientation refers to an individual’s tendency to consider the future rather than the immediate consequences of one’s current behaviors, and it also reflects how much the individual is able to control the impulse of immediate happiness (Strathman et al., 1994). People with future orientation would prefer to consider future consequences and to delay gratification into the future, rather than pursue immediate pleasurable activities (Prenda and Lachman, 2001). Employees with future orientation are willing to sacrifice immediate happiness to achieve their long-term goals (Barber et al., 2009; Steinberg et al., 2009; Gick, 2014). To some extent, employees’ future orientation reflects the extent of their autonomy.

According to the definition and previous studies on cyberloafing behaviors, many employees do not concern with their future, and waste their working hours escaping their work. Thus, cyberloafing behaviors is a form of procrastination (Lavoie and Pychyl, 2001), which is a learned habit that develops from a human preference for pleasurable activities and short-term reward (Haycock et al., 1998). Ferrari and Diaz-Morales (2007) found that procrastination was positively associated with present-hedonist, but negatively with future time orientations. Cyberloafing behaviors are an example of aimless behaviors (Kim and Byrne, 2011), which distract employees from their work. People with high future orientation proactively consider the future consequences of their current behaviors and plan ahead for their future work and life.

Based on the above information, we suspect that an individual’s future orientation and cyberloafing behaviors can link between each other robustly. Employees who hold high future orientation tend to have less cyberloafing behaviors compared to those who hold low future orientation (Hypothesis 1).

Furthermore, there are two categories of individual future orientation: trait future orientation (Gjesme, 1976, 1979a,b) and situational future orientation (Halvari, 1991; Zaleski, 1992). Therefore, we tested the dampening effect of employees’ future orientation on their cyberloafing behaviors using a self-report questionnaire survey (Study 1) and a priming experiment (Study 2).

### Self-control as Mediator

If Hypotheses 1 is valid, then the psychological processes underlying the dampening effect of employees’ future orientation on their cyberloafing must be further explained. Gottfredson’s theory suggested that individual problem behaviors were induced through a lack of self-control (Gottfredson and Hirschi, 1990). Self-control is defined as “the ability to monitor, inhibit, persevere and adapt behavior, emotions, thoughts, and desires to achieve a certain goal” (Moffitt et al., 2011). Some researches have shown that trait future orientation was positively associated with an individual’s self-control. Individuals with high future orientation prefer to consider future consequences and to delay gratification into the future (Prenda and Lachman, 2001). The theory of delayed gratification reveals that people with good delayed gratification would have the strong self-control abilities needed to inhibit prioritizing immediate gratification (Funder et al., 1983; Funder and Block, 1989; Krueger et al., 1996). Some other research has shown that making a plan for the future increases one’s self-control abilities (Prenda and Lachman, 2001; Azizli et al., 2015). Planning for the future by oneself gives people a feeling of autonomy, which can enhance self-control (Muraven et al., 2008). Individual differences in future time perspectives are important for developing self-control and are positively related to self-control (Joireman et al., 2006; Romer et al., 2010). All of these studies have revealed that individuals’ future orientation is positively associated with their self-control.

In addition, Kim and Byrne (2011) suggested that cyberloafing behaviors are caused by a lack of self-control. Rahimnia and Mazidi (2015) found that employees’ self-control was negatively associated with their cyberloafing. There are also many empirical studies supporting self-control as a protective factor against problematic Internet use (Kim et al., 2008; Li et al., 2013). Furthermore, the ego depletion model of cyberloafing suggests that when an employee is drained of self-control resources, he or she is likely to engage in cyberloafing (Baumeister et al., 2000; Wagner et al., 2012). Thus, an individual’s self-control is negatively associated with their cyberloafing behaviors.

According to the Skinner’s (1996) view, the construct of self-control can be divided into objective self-control and subjective self-control. The former one refers to a kind of actual self-control ability, and the latter one refers to a sense of self-control individuals perceived. Besides examining the existing theoretical and empirical evidence (Joireman et al., 2008; Restubog et al., 2011), the present study primarily focuses on the role of objective and subjective self-control and constructs a mediation model to establish a research precedent for its importance in linking future orientation and cyberloafing. Given the evidence presented above, we hypothesize that employees’ self-control plays a mediating role between future orientation and cyberloafing behaviors (Hypothesis 2). An individual’s future orientation leads to high self-control, which in turn reduces their cyberloafing behaviors.

### Overview of the Current Studies

Based on literature review of previous studies, we propose the following two hypotheses:

Hypothesis 1: employees’ future orientation is negatively associated with their cyberloafing behaviors; and
Hypothesis 2: employees’ self-control plays a mediating role in the relationship between future orientation and cyberloafing behaviors.

We conducted two studies to test Hypothesis 1 and 2. In Study 1, a series of questionnaires was used to explore the correlation between employees’ trait future orientation and their cyberloafing behaviors, and to test whether their objective self-control was a potential mediator between future orientation and cyberloafing behaviors. In Study 2, we did a priming experiment to further demonstrate whether employees’ situational future orientation could dampen their cyberloafing behaviors by the mediating role of their subjective self-control, and constructed a mediation model to confirm the hypotheses.

## Study 1

The objective of Study 1 was twofold. Firstly, we explored whether chronic differences in future orientation could predict employees’ cyberloafing behaviors. We expected that employees’ future orientation was negatively associated with the frequency of their cyberloafing behaviors. Secondly, we examined whether employees’ objective self-control can mediate the dampening effect of employees’ future orientation on cyberloafing behaviors or not. We predicted that employees’ self-control mediates the relationship between their future orientation and cyberloafing behaviors.

### Methods

#### Participants

A total of 232 employees completed a series of questionnaires online or by paper and pencil. The final valid sample size is 210 participants (68 males and 142 females). Twenty two participants were invalid, among them six participants came from school, five participants came from public institution or government agency, and 12 participants didn’t seriously complete questionnaires. The effective rate of sample is 90.52%. The average age of the 210 participants was 28.11 years (*SD* = 5.34), with a range from 19 to 55 years. The participants had different career backgrounds, and they all worked for different enterprises which explicitly prohibit the use of the internet for non-work purposes. To be eligible for the study, all the participants are able to access the Internet at work, and on average, the time they spent surfing the Internet on personal computers and mobile devices was 5.72 h (*SD* = 2.61) per workday.

#### Procedures

Participants completed a series of questionnaires online or by paper and pencil. To be more specific, after filling in demographic information, all of the participants completed a consideration of future consequences scale, a self-control scale, and a measure of cyberloafing behaviors.

#### Measures

##### Consideration of future consequences scale (CFC)

Employees’ future orientation was measured by the CFC scale, which contains 12 general statements (e.g., “I am willing to sacrifice my immediate happiness or well-being in order to achieve future outcomes.”; “I think that sacrificing now is usually unnecessary since future outcomes can be dealt with at a later time.”) reflecting an individual tendency to consider the future consequences of his/her behavior (Strathman et al., 1994; Joireman et al., 2008). There were five statements (CFC-future) reflecting the consideration of future consequences, and the remaining seven statements (CFC-immediate) were reverse scored. Each item uses a five-point Likert scale assessing to what extent each item is characteristic of the individual ranging from 1 (very uncharacteristic) to 5 (very characteristic). The sum of these items, with appropriate reversals, composes the future orientation score. The higher aggregated score means higher levels of future orientation. The Cronbach’s alpha coefficient was 0.75 for the present sample.

##### Self-control scale

The Brief Self-Control Scale (Tangney et al., 2004) was used to measure employees’ trait self-control ability, which contains 13 items pertaining to control over thoughts, emotion control, impulse control, performance regulation, and habit breaking. Two example questions are “I am good at resisting temptation” and “People would say that I have iron self-discipline.” Responses are indicated on a five-point Likert scale, ranging from 1 (not at all like me) to 5 (very much like me). The higher aggregated score indicates higher levels of employees’ trait self-control. The Cronbach’s alpha coefficient was 0.70.

##### Cyberloafing

Cyberloafing behaviors were measured using a 19-item scale developed from a version of Lim’s (2002) cyberloafing scale (Lim, 2002; Blanchard and Henle, 2008; Askew et al., 2014). A representative item is “Sent non-work related email.” Participants rate the frequency they were engaged in a list of cyberloafing behaviors on a 5-point scale (1 = Never, 3 = Occasionally, and 5 = Constantly). Higher aggregated scores represent being more frequently engaged in actual cyberloafing behaviors. The Cronbach’s alpha coefficient was 0.88.

### Results

#### Descriptive Analyses

Means, standard deviations and correlation coefficients between the major variables are presented in **Table 1**. The results indicate that there are significant correlations between any pair of CFC, self-control, and cyberloafing behaviors. To be more specific, CFC is positively related to self-control (*r* = 0.33, *p* < 0.001) and negatively related to cyberloafing (*r* = –0.25, *p* < 0.001). Additionally, self-control is negatively correlated with cyberloafing (*r* = –0.28, *p* < 0.001).

**Table 1:** Descriptive statistics and intercorrelations between the variables.

Variables	*M*	*SD*	1	2	3	4	5
(1) Gender	0.32	0.47	–				
(2) Age	28.11	5.34	0.056	–			
(3) CFC	38.89	6.01	0.069	0.027	–		
(4) Self-control	39.45	5.19	-0.007	0.25^∗∗∗^	0.33^∗∗∗^	–	
(5) Cyberloafing	45.16	11.45	0.069	-0.061	-0.25^∗∗∗^	-0.28^∗∗∗^	–

*Gender was dummy coded such that 0 = “female” and 1 = “male”. ^∗∗∗^*p* < 0.001.*

#### Testing the Mediating Role of Self-control in the Relationship between CFC and Cyberloafing

To explain the psychological processes underlying the dampening effect of employees’ future orientation on their cyberloafing behaviors. We tested the mediation effect of self-control on the relationship between employees’ CFC and cyberloafing using multiple regression analyses. According to Baron and Kenny (1986), the following effects should be present to establish a mediation effect: (a) a significant effect of employees’ CFC on cyberloafing; (b) a significant effect of CFC on self-control; (c) a noteworthy relationship between self-control and cyberloafing when CFC is controlled for; and (d) a noteworthy reduction of the effect of CFC on cyberloafing when self-control is included in the model. After adjusting for age and gender, self-control is found to mediate the associations between the employees’ CFC and cyberloafing with the following patterns:

Employees’ CFC is negatively associated with cyberloafing (see equation 1 of **Table 2**, *B* = –0.49, *p* < 0.01) and positively associated with self-control (see equation 2 of **Table 2**, *B* = 0.25, *p* < 0.001). Equation 3 in **Table 2** shows that employees’ self-control can significantly predict cyberloafing (*B* = –0.52, *p* < 0.01). These results indicate that the mediation model is established.

**Table 2:** Test the mediation effects of self-control on cyberloafing (*N* = 210).

Predictors	Equation 1			Equation 2			Equation 3		
	(criterion: cyberloafing)			(criterion: self-control)			(criterion: cyberloafing)		
	*B*	95%CI	β	*B*	95%CI	β	*B*	95%CI	β
Gender	2.69	(-0.98, 6.37)	0.10	-0.42	(-1.97, 1.14)	-0.04	2.48	(-1.12, 6.08)	0.096
Age	-0.22	(-0.54, 0.10)	-0.098	0.18	(0.049, 0.32)	0.19^∗∗^	-0.13	(-0.45, 0.19)	-0.056
CFC	-0.49	(-0.77, -0.21)	-0.25^∗∗^	0.25	(0.13, 0.37)	0.29^∗∗∗^	-0.36	(-0.65, -0.07)	-0.18^∗^
Self-control							-0.52	(-0.86, -0.17)	-0.22^∗∗^
*R^2^*		0.08^∗∗^			0.13^∗∗∗^			0.13^∗∗∗^	

*Each column set is a regression equation that predicts the criterion at the top of the column. Gender was dummy coded such that 0 = “female” and 1 = “male”. ^∗^*p* < 0.05; ^∗∗^*p* < 0.01; ^∗∗∗^*p* < 0.001.*

**FIGURE 1:**
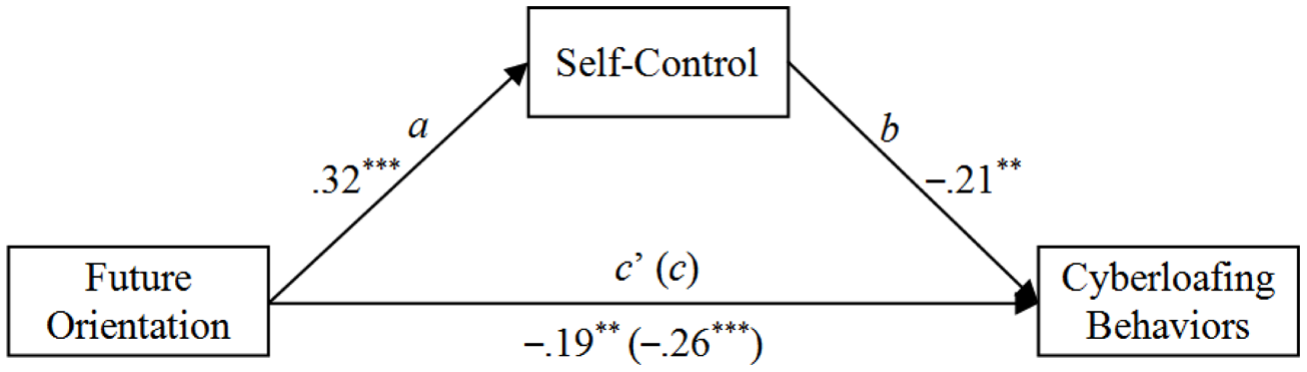
**Mediation of future orientation and cyberloafing behaviors by self-control.** Total effect (*c*): Effect = –0.26, *SE* = 0.07, *p* < 0.001, LLCI = -0.39, ULCI = -0.13; Direct effect (*c*’): Effect = -0.19, *SE* = 0.07, *P* < 0.01, LLCI = -0.33, ULCI = -0.05; Indirect effect (*ab*): Effect = -0.07, Boot *SE* = 0.03, Boot LLCI = -0.14, Boot ULCI = -0.02. To yield standardized coefficients, all variables were converted to *z*-scores prior to analysis. ^∗∗^*p* < 0.01; ^∗∗∗^*p* < 0.001.

We also conducted regression analyses according to the specification set out by Andrew Hayes’ (2013) PROCESS for SPSS using model 4 (a bootstrapping CI method with *N* = 5000 bootstrap samples) to further verify the mediation model. As illustrated in **Figure 1**, after controlling gender and age, CFC was associated with cyberloafing, and this relationship was partially mediated by self-control.

### Discussion

The results of Study 1 demonstrate that chronic differences in future orientation could predict employees’ cyberloafing behaviors. The more future consequences employees consider, the less cyberloafing behaviors they get engaged in during working hours. The results also show that employees’ objective self-control mediates the relationship between their future orientation and cyberloafing behaviors. At the personality trait level, all these results confirmed Hypotheses 1 and 2.

However, Study 1 was correlational: although we demonstrated that the relationship between employees’ chronic future orientation and cyberloafing is negative, the results cannot be the basis for conclusions about causality. If employees make a plan for their future, would this plan dampen their intentions to engage in cyberloafing and reduce their attitudes toward cyberloafing behaviors? We, therefore, conducted Study 2 in which we experimentally primed employees’ future orientation and measured the dampening effect on cyberloafing.

## Study 2

Study 2 has two goals. The first is to explore whether the situational priming future orientation can reduce employees’ cyberloafing attitudes and intentions. The second is to replicate the mediation effect of self-control on the relationship between employees’ future orientation and cyberloafing. We expected that the future orientation priming would dampen employees’ intentions to engage in cyberloafing behaviors during working hours, and the sense of self control that they perceived (subjective self-control) would mediate this dampening effect.

### Methods

#### Participants

This study was completed by another 46 participants (31 females and 15 males). They were full-time employees in different enterprises which explicitly prohibit the use of the internet for non-work purposes and their average age was 27.91 (*SD* = 5.86). Participants joined the study voluntarily and were given a pen or a notebook as gifts for participation.

#### Procedures

Prior to the research, ethical approval was obtained from the Committee of Protection of Subjects at Beijing Normal University. All participants were required to read and approve the informed consent before participating in this research.

This study included two parts. First, a between-subjects design was adopted and participants were randomly assigned to one of two conditions: the future orientation condition (*N* = 24) or the control condition (*N* = 22). **In the future orientation condition**, a priming paradigm was used to induce employees’ future orientation (Gjesme, 1983; Prenda and Lachman, 2001). The participants’ task was, “*Please make a five years career plan for yourself, write down what you want to be and how you will fulfill your plan in fifteen minutes.*” **While in the control condition**, the task was “*Please record all the things you had seen or heard on today, and write them down on the paper in fifteen minutes.*” All participants were given a sheet of paper with 16 lines to complete this task.

In the second part of Study 2, participants were asked to complete measures of the CFC-general scale (four items), the Positive and Negative Affect Schedule (PANAS), the Sense of Control Scale, and measures of cyberloafing attitudes and intentions.

#### Measures

All the measurement tools that were originally developed in foreign languages were translated into Chinese using back-translation procedures, and their validity and reliability were examined.

##### CFC-general

The CFC-general is a short-form of the original CFC scale (Strathman et al., 1994; Joireman et al., 2008) that was used in Study 1. The CFC-general contains four items (item 1, 2, 10, and 11) with the two highest factor-loading in each factor were extracted from the original CFC’s item pool. These four items were used to check the manipulation of employees’ future orientation in previous research (van Beek et al., 2013). These items were based on a 4-point Likert scale: 1 = not at all, 2 = a little, 3 = some, and 4 = a lot. Participants were asked to indicate to what extent each item described them, and the maximum score was 16. In the current study, we obtained Cronbach’s alpha coefficients of 0.82 and 0.70 for the CFC-future factor (items 1 and 2) and the CFC-immediate factor (items 10 and 11), respectively. The sum of these four items, with appropriate reversal, made up the future orientation score (CFC-general). Higher scores on the CFC-general indicated that the employees were more concerned about future orientation.

##### Positive and negative affect schedule

The PANAS was used as a filler task to determine whether the priming triggered any unwanted positive or negative affective reaction. The PANAS consists of two subsets of items, one contains 10 items measuring positive items (e.g., “interesting”) and the other contains 10 different items measuring negative items (e.g., “shame”) affect (Watson et al., 1988). Scores from the two subsets are aggregated separately to represent the positive and negative affects. Both subscales were averaged to form reliable scales (Cronbach’s α = 0.90 and 0.87).

##### Sense of personal control scale

The personal mastery scale (Pearlin and Schooler, 1978) was widely used to estimate individual’s sense of personal control (Lachman and Weaver, 1998; Ross and Broh, 2000). We used that scale to measure the sense of self-control employees perceived. It contains four items (e.g., “I can do just about anything I really set my mind to” and “What happens to me in the future mostly depends on me”). Participants indicated their responses to these four items on a 7-point Likert scale with end points 1 (strongly disagree) and 7 (strongly agree). The higher aggregated score indicates higher employees’ perceived self-control ability. The Cronbach’s alpha coefficient was 0.69.

##### Cyberloafing attitudes

The attitudes scale (Ajzen, 2006; Askew et al., 2014) consists of four items asking participants to rate the extent to which they think cyberloafing is *valuable, enjoyable, beneficial*, and *good*. The four items were rated on a seven-point scale ranging from 1 (extremely worthless, unenjoyable, harmful, and bad) to 7 (extremely valuable, enjoyable, beneficial, and good). Lower scores indicate that employees held less positive attitudes toward Internet use at work for personal reasons. The Cronbach’s alpha coefficient was 0.82.

##### Cyberloafing intentions

The intentions scale (Ajzen, 2006; Askew et al., 2014) consists of six items asking participants to rate their intentions to engage in six common cyberloafing behaviors in the forthcoming month (e.g., I intend to send a non-work related email at least once in the forthcoming month). The items were rated on a seven-point scale ranging from 1 = extremely no intention to 7 = extremely intention. Higher scores indicate that the employee has higher intentions to engage in cyberloafing behaviors in the forthcoming month. The Cronbach’s alpha coefficient was 0.87.

### Results

#### Manipulation Check

To assess the effectiveness of the future orientation priming manipulation, we conducted an independent sample *t*-test for the CFC-future, the CFC-immediate, the CFC-general, and the PANAS. The analyses revealed that participants in the future orientation priming condition (*M* = 5.29, *SD* = 1.60) focus more on the future consequences than those in the control condition (*M* = 4.05, *SD* = 1.59), *t* = 2.41, *p* < 0.05, Cohen’s *d* = 0.73, but there was no significant difference on the immediate consequence (*t* = –1.06, *p* > 0.05, Cohen’s *d* = –0.31). In addition, the analyses did not identify any significant effects of our manipulations on the positive and negative subscale scores of the PANAS, which were all significant (2-tailed) > 0.05. The above results suggest that our manipulation of future orientation is successful.

#### The Influence of Employees’ Future Orientation on Cyberloafing

A *t*-test analysis was conducted to explore whether employees’ future orientations affected their cyberloafing attitudes and intentions. Participants primed with a future orientation were more concerned about avoiding cyberloafing attitudes (*M* = 4.06, *SD* = 0.91) and intentions (*M* = 3.67, *SD* = 0.90) than the employees in the control group (attitudes: *M* = 5.22, *SD* = 0.92; intentions: *M* = 5.52, *SD* = 1.37). Employees in the future orientation condition had more negative attitudes (*t* = –4.27, *p* < 0.001, Cohen’s *d* = –1.29) and lower intentions (*t* = –5.45, *p* < 0.001, Cohen’s *d* = –1.60) toward cyberloafing. Employees in the future orientation condition (*M* = 5.27, *SD* = 0.60) also perceived more self-control ability than those in the control condition (*M* = 4.77, *SD* = 0.88; *t* = 2.26, *p* < 0.05, Cohen’s *d* = 0.68). These results are presented in **Table 3**.

**Table 3:** A *t*-test analysis of the future orientation condition and the control condition.

	Future orientation condition(*N* = 24)		Control condition (*N* = 22)		*t*	Cohen’s *d*
	*M*	*SD*	*M*	*SD*		
Gender	0.38	0.49	0.27	0.46	0.73	0.22
Age	28.00	5.28	27.82	6.57	0.10	0.03
Years of working	6.79	4.79	6.55	4.93	0.17	0.05
Positive affect	30.67	7.47	28.18	7.70	1.11	0.33
Negative affect	19.54	5.95	18.18	6.51	0.74	0.22
CFC-future	5.29	1.60	4.05	1.59	2.65^∗^	0.78
CFC-immediate	3.58	1.38	4.09	1.85	-1.06	-0.31
CFC-general	11.71	2.24	9.95	2.70	2.41^∗^	0.73
Sense of control	5.27	0.60	4.77	0.88	2.26^∗^	0.68
Cyberloafing attitudes	4.06	0.91	5.22	0.92	-4.27^∗∗∗^	-1.29
Cyberloafing intentions	3.67	0.90	5.52	1.37	-5.45^∗∗∗^	-1.60

*Gender was dummy coded such that 0 = “female” and 1 = “male”. ^∗^*p* < 0.05; ^∗∗∗^*p* < 0.001.*

#### Mediation Analyses

We further explored the mediating role of sense of self-control on the relationships between employees’ future orientation and cyberloafing intentions. A series of regression equations relating future orientation (the independent variable), sense of self-control (the potential mediator), and cyberloafing intentions (the dependent variable) were performed using the same analysis performed in Study 1. The results of this analysis replicated the findings of Study 1.

Employees’ future orientation can negatively predict their cyberloafing intentions (see equation 1 of **Table 4**, β = –0.63, *p* < 0.001) and positively predict the sense of self-control they perceived (see equation 2 of **Table 4**, β = 0.49, *p* < 0.05). From equation 3 in **Table 4**, the sense of self-control employees perceived can significantly predict cyberloafing (β = –0.51, *p* < 0.05). The results show that the mediation model is established.

**Table 4:** A test of the mediation effects of self-control on cyberloafing intentions (*N* = 46).

Predictors	Equation 1			Equation 2			Equation 3		
	(criterion: cyberloafing intentions)			(criterion: sense of control)			(criterion: cyberloafing intentions)		
	*B*	95% CI	β	*B*	95% CI	β	*B*	95% CI	β
Gender	-0.36	(-1.11, 0.38)	-0.12	0.15	(-0.34, 0.64)	0.091	-0.29	(-1.00, 0.42)	-0.093
Age	0.049	(-0.011, 0.11)	0.19	-0.012	(-0.052, 0.027)	-0.093	0.042	(-0.015, 0.099)	0.17
Future orientation	-1.82	(-2.50, -1.14)	-0.63^∗∗∗^	0.49	(0.031, 0.94)	0.31^∗^	-1.57	(-2.26, -0.89)	-0.54^∗∗∗^
Sense of self-Control							-0.51	(-0.95, -0.66)	-0.27^∗^
*R^2^*		0.44^∗∗∗^			0.12^∗^			0.51^∗∗∗^	

*Each column set is a regression equation that predicts the criterion at the top of the column. Gender was dummy coded such that 0 = “female” and 1 = “male.” Future Orientation was also dummy coded such that 0 = “control condition” and 1 = “future priming condition.” ^∗^*p* < 0.05; ^∗∗∗^*p* < 0.001.*

**FIGURE 2:**
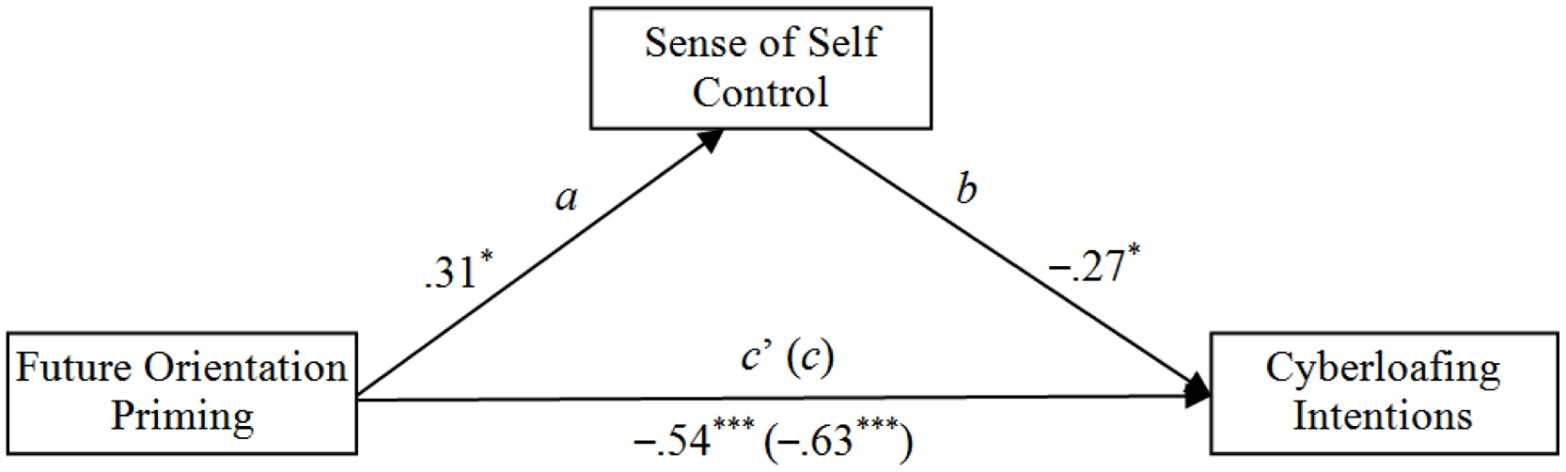
**Results from the mediation analyses testing sense of self control as a mediator of the dampening effect of employees’ future orientation on cyberloafing intentions.** Total effect (c): Effect = -0.63, *SE* = 0.12, *p* < 0.001, LLCI = -0.86, ULCI = -0.39; Direct effect (c’): Effect = -0.54, *SE* = 0.12, *P* < 0.01, LLCI = -0.77, ULCI = -0.31; Indirect effect (ab): Effect = -0.09, Boot *SE* = 0.05, Boot LLCI = -0.22, Boot ULCI = -0.01. To yield standardized coefficients, all variables were converted to *z*-scores prior to analysis. ^∗^*p* < 0.05; ^∗∗∗^*p* < 0.001.

To further verify the mediation model, we again used the PROCESS macro for SPSS (Model 4; Hayes, 2013). PROCESS calculates a bias-corrected and accelerated bootstrapped confidence interval (*N* = 5000 bootstrap samples) for the size of each indirect effect, with significant mediation indicated by a confidence interval that does not contain zero. Our results are illustrated in **Figure 2**. The results indicate that the dampening effect of employees’ future orientation on cyberloafing intentions is partially mediated by the sense of self-control employees perceived.

### Discussion

The results of Study 2 show that when employees plan for the future by themselves, they are more likely to focus on future consequences and to have higher future orientation. The higher future orientation promotes employees to perceive higher sense of self-control, and decreases their attitudes and intentions related to cyberloafing behaviors during working hours. Additionally, the mediation model reveals that employees’ sense of self-control mediate the dampening effect of future orientation on their cyberloafing intentions. Previous studies have suggested that employees’ cyberloafing intentions can predict their actual behaviors (Askew et al., 2014). The findings of Study 2 replicate the results of Study 1, and thus support Hypotheses 1 and 2.

## General Discussion

Confucius, the great ancient Chinese philosopher, once said “Think long, or worries are not far away.” This wisdom statement means that “If a man does not plan for the future, he will be distracted by what happens in the short term.” For employees or their enterprises, cyberloafing behaviors are short-sighted behaviors, which may lead to trouble for employees and their enterprises. Our findings are consistent with the implications of this sentence. We find that employees who are more future-oriented (“think long”) tend to have fewer cyberloafing behaviors (“worries”) during their work time. We used both a series of self-report measures (Study 1) and a priming paradigm (Study 2) to confirm the inference that employees’ future orientation (an important individual factor) would dampen their cyberloafing behaviors (Hypothesis 1) and that employees’ self-control mediates the dampening effect (Hypothesis 2). Hypotheses 1 and 2 were confirmed in both Study 1 (trait future orientation) and Study 2 (situational future orientation).

Our findings reveal that both trait and situational future orientation can reduce employees’ cyberloafing. To date, companies and employers have used external demands to reduce employees’ cyberloafing behaviors (Blanchard and Henle, 2008; Askew, 2012; RuningSawitri, 2012; Ugrin and Pearson, 2013). However, we wanted to test a way to reduce employees’ cyberloafing behaviors from the perspective of individual autonomy. In our two studies, these two types of future orientation reflect employees’ autonomy as they are not passively manipulated by external requirements. People could proactively set a long-term goal for themselves by considering the future consequences of their current behaviors and then making a plan for their future. They would then develop intrinsic motivation to realize their future goal. According to the SDT (Gagne and Deci, 2005), people would wholly volitionally reduce their pleasure activities (e.g., cyberloafing behaviors) to strive toward their future goal. The results of our studies confirm the explanation of SDT. Our results show that employees who tend to proactively consider future consequences and engage in less cyberloafing behaviors. When employees make a plan for their future by themselves, they reduce their attitudes and intentions toward cyberloafing behaviors.

Our findings also offer an insight into the underlying psychological mechanism of the dampening effect of employees’ future orientation on cyberloafing behaviors. The results of mediation analyses reveal that employees’ self-control mediate the dampening effect. Our studies have confirmed that employee’s future orientation can positively influence their self-control, which, in turn, reduces their cyberloafing behaviors. In Study 1, we find that employee’s trait future orientation is positively associated with their self-control. Moreover, the results of Study 2 show that priming employees’ future orientation also can enhance their sense of self-control. It is important to note that the self-control in our Study 1 and 2 are different. Previous researches indicated that the control can be divided into objective and subjective control (Skinner, 1996). In Study 1, the self-control is a kind of objective control, which refers to an actual control ability (Skinner, 1996). This kind of self-control is relatively stable control ability, which needs a long time to improve (Kirschenbaum et al., 1981). Previous evidences showed that developing employees’ self-control abilities can reduce their cyberloafing behaviors (Rahimnia and Mazidi, 2015). In addition, Gottfredson and Hirschi (1990) suggested that individual’s problem behaviors were induced by a lack of self-control ability. Cyberloafing is one of the most acknowledged problematic internet use behaviors in the workplace (Lim, 2002; Blanchard and Henle, 2008). Therefore, from the personality trait perspective, we are convinced that employees’ objective self-control ability can mediate the dampening effect of employees’ trait future orientation on their cyeberloafing behaviors. The findings of study 1 confirm the Hypothesis 1 and 2.

In Study 2, the self-control employees perceived belongs to subjective control, which refers to an individual’s beliefs about how much control is available (Skinner, 1996). People perceived higher sense of personal control would have stronger belief that they can control themselves and external environment factors to overcome all kinds of problems in their life (Skinner, 1996; Ross and Broh, 2000). The results of our experiment suggest that priming employees with a future-oriented planning would increase their sense of control. These findings are consistent with those from previous studies (Prenda and Lachman, 2001). Moreover, our results also reveal that employees have less intention to engage in cyberloafing behaviors when they perceived higher sense of self-control. From the perspective of SDT (Baard et al., 2004; Gagne and Deci, 2005), when employees determine their own future they develop a sense of autonomy and self-control, which decrease employees’ intentions to act cyberloafing behaviors. It is worth noting that the self-control in Study 2 is not actual self-control, but a sense of personal control. Many theorists are convinced that perceived personal control is a more powerful predictor of functioning than actual control (Averill, 1973; Burger, 1989). Therefore, the relationship between employees’ situational future orientation and their cyberloafing intentions can be mediated by the sense of self-control employees perceived. The findings of Study 2 replicate the results of Study 1, and thus confirm the hypotheses 1 and 2.

### Implications

This research extends the prior work on cyberloafing behaviors by highlighting the influence of individual factors. Being different from previous research, which has emphasized the external environment factors that affect cyberloafing, our findings illuminate how employees’ future orientation can reduce cyberloafing behaviors through self-control. In previous studies, employees are subject to external demands to reduce their cyberloafing behaviors. However, we find that employees’ cyberloafing behaviors also can be reduced by themselves. Our results indicate that employees can dampen their cyberloafing behaviors by considering the future consequences and by making a plan for their future.

The present research also provides many practical implications. Particularly, our findings suggest that managers can effect individual’s future orientation and self-control to reduce employees’ cyberloafing behaviors. For instance, we propose two methods for reducing employees’ cyberloafing behaviors (Strathman et al., 1994; Prenda and Lachman, 2001; Barber et al., 2009). First, enterprises could train their employees to create a long term career plan in support of their life goals. Second, enterprises can improve employees’ self-control, or can develop their self-regulation skills (Vandellen et al., 2012). Our results provide the theoretical basis for formulating specific training programs to reduce employees’ cyberloafing behaviors.

### Limitations and Directions for Future Research

Several limitations should be considered when interpreting the results of the present study. First, it is important to note that although self-report measurement is widely used and the instruments employed in Study 1 and 2 have a good reliability, the common-method bias is still inevitable. Future research can benefit from using multiple informants and multiple data collection methods simultaneously. Second, self-control functioned as a partial mediator in this study, which implies the existence of other pathways, such as employee’s achievement motives (Woo et al., 2007). The path from future orientation to employees’ cyberloafing behaviors can be very complex and requires further exploration. Future research could explore additional potential mediators of the relationship between employees’ future orientation and their cyberloafing behaviors. Third, to be sure, we did not actually measure employees’ cyberloafing behaviors but intentions toward cyberloafing in Study 2. Previous studies have revealed that individuals’ cyberloafing intentions can predict their actual cyberloafing behaviors (Askew et al., 2014). However, future research should measure employees’ actual cyberloafing behaviors in the work place.

## Conclusion

The two studies expand upon the existing knowledge about cyberloafing behaviors, and the findings are novel and insightful both theoretically and practically. From the perspective of individual factors, this research not only clarifies that employee’s future orientations are negatively associated with their cyberloafing behaviors, but also supports the role of their self-control (objective and subjective) as a mediator in this relationship. In short, the results suggest that employees’ self-control mediates the dampening effect of their future orientation on cyberloafing behaviors. To this end, the present study offers a valuable foundation for future work.

## Conflict of Interest Statement

The authors declare that the research was conducted in the absence of any commercial or financial relationships that could be construed as a potential conflict of interest.
